# Does e-shopping service quality enhance customers’ e-shopping adoption? An extended perspective of unified theory of acceptance and use of technology

**DOI:** 10.1371/journal.pone.0263652

**Published:** 2022-02-25

**Authors:** Amjad Ur Rehman, Shahid Bashir, Asif Mahmood, Haroon Karim, Zameer Nawaz

**Affiliations:** 1 Department of Business Studies, Al-Qadir College, Sohawa District Jhelum, Pakistan; 2 Department of Business Studies, Namal University, Mianwali, Pakistan; St John’s University, UNITED KINGDOM

## Abstract

This research advances the knowledge in customer behavior literature by adding new exogenous and moderating variables to the UTAUT framework. It explores the relationships among e-shopping service quality (an exogenous variable), e-shopping drivers (performance expectancy, effort expectation, social influence, and facilitating conditions), e-shopping intention, and e-shopping adoption with the moderating role of offline brand trust in an e-shopping context. Structure equation modeling was performed to confirm the distinctiveness of variables and path analysis based on a sample size of 356 e-shoppers in Pakistan. The outcomes demonstrate that e-shopping drivers are influenced by e-shopping service quality. Moreover, e-shopping intention and e-shopping adoption are led by e-shopping drivers. Furthermore, the relationship between e-shopping drivers and e-shopping intention is moderated by offline brand trust. The discussion of theoretical and practical implications and study limitations are also presented.

## 1. Introduction

The rapid evolution of e-commerce or e-business offers new frameworks (or models) for online businesses worldwide [1]. Accordingly, there is an exponential development in this sector; for example, the online retailing market is expected to rise from $24 billion (United States currency) in 2019 to $98 billion in 2024 [2]. However, with this rapid upsurge, e-businesses face challenges as well, specifically in the commercial segment, to market the product(s) and service(s) through a new channel of distribution (e-shopping) [3,4]. Although several researchers, e.g., [5–7], proposed e-shopping antecedents, various scholars such as [7,8] have recognized that the e-shopping phenomenon is still underdeveloped. Consequently, comprehensive research of such factors would help understand consumer behavior.

The author studied the user’s technology acceptance-related literature well to compare prominent frameworks [9]. They eventually developed a unified framework named Unified Theory of Acceptance and Use of Technology (UTAUT). Several core drivers, such as facilitating conditions, societal influence, and personal expectancy (performance and effort), are linked to UTAUT. Authors have assessed this framework mainly in the context of e-shopping [9]. Later, authors have extended UTAUT (named UTAUT 2) by exploring the technological internet usage of mobiles [10].

Researchers also emphasized and suggested further extensions also, for instance, the addition of new exogenous variable(s), endogenous(s)/driver(s), moderation mechanism(s) or outcome variable(s) to the theory [11]. Literature also observed some shortcomings in the UTAUT; according to them, buyers’ acquisition and long-term retention can be inhabited by dealing with the elements of online presence in e-shopping [12]. Therefore, it is essential to know how the sellers build a sense of presence through service quality. For instance, web quality may affect buyer’s perceptions and behaviors [13], improve the e-shopping experience [14,15], and enable e-interactions [16]. Consequently, the quality of websites could boost buyers’ loyalty to the sellers [17]. Since the quality of a website can influence making an online presence, it can be valuable to explore the influence of e-shopping service quality (or online shopping service quality) as an additional construct of UTAUT [12,18].

Similarly, trust is also an essential element in interacting with retailers and customers and guaranteeing future interactions with the brand [19,20]. It often reduces risk perceptions’ negative impacts and ambiguities [19,21,22]. The researchers e.g., [18] have added to UTAUT in different contexts and suggested further research, for example, to comprehensively investigate the offline brand trust in consumer technology usage. In this regard, researchers have made an effort to examine the function of offline brand trust as a moderator (as an additional construct of UTAUT) between e-shopping drivers and e-shopping intention [18]. Although the study has confirmed no such function of offline brand trust as a moderator, it was observed to have several restrictions that give a good reason for advanced research in this domain. First, the authors have used only two e-shopping drivers (i.e., performance expectancy and effort expectancy) in their study, leaving a gap to study two other e-shopping drivers, i.e., facilitating conditions, societal influence. Second, the authors have surveyed the university students of Pakistan; whereas, according to one recent report (published in Business Recorder), above half of the e-shoppers (56%) in Pakistan are aged between 25 and 34 [23]. Such age group mainly covers early or mid-career people, not university students. Therefore, the university students might not fully cover or justify the entire sample frame. Last, the authors have used a sample size of 167 to develop their structural equation model, whereas several researchers have suggested considering an even larger sample size (e.g., 200) [24–26]. Consequently, to gather more efficient research outcomes, there is a need to re-examine the function of offline brand trust as a moderator between e-shopping drivers and e-shopping intention.

Since advancements in the knowledge of customer behavior literature through adding new exogenous and moderating variables to the UTAUT framework is a relatively unsearched area, a study endeavor can be unique and beneficial. Therefore, grounded on the mentioned rationales, our aim is to explore the relationships among e-shopping service quality (an exogenous variable), e-shopping drivers (performance expectancy, effort expectation, social influence, and facilitating conditions), e-shopping intention, and e-shopping adoption with the moderating role of offline brand trust in an e-shopping context.

In this context, this study will facilitate the readers to understand e-shopping adoption better and determine the most critical factors that can predict customers’ decisions about e-shopping. The framework of this study would conceptualize those important features that online customers potentially consider. This study would make many theoretical and practical implications by adding value to the existing literature and e-shopping market. It would extend the theory (UTAUT) by incorporating a new exogenous variable (e-shopping service quality), and a new moderator (offline brand trust). It would also primarily help online retailers and marketers to form their policies, plans, and frameworks to increase the overall efficiency of the e-shopping market.

Moreover, this study would help improve the service quality of online shopping to fulfill the customers’ satisfaction requirements. Furthermore, since the survey data have been collected from Pakistan, it can extensively be considered a basis to understand the e-shopping behavior of South Asian consumers. It is the area with around 25% population of the entire world, where the consumers are rapidly shifting from offline to online compared to the Western countries.

In the following section, the literature will be reviewed, and the hypotheses will be proposed. Afterward, the research methodology will be explained, and the empirical outcomes of the proposed hypotheses will be examined and discussed. Lastly, both theoretical and applied implications will be presented.

## 2. Unified Theory of Acceptance and Use of Technology (UTAUT)

Researchers developed the unified theory of acceptance and use of technology (UTAUT) as a technology acceptance model [10]. The UTAUT seeks to describe how users want to use an information system and how they actually utilize it [9]. Since the development of UTAUT, it has been empirically tested and confirmed as an advanced theory to explain e-shopping behavior [27].

UTAUT has four antecedent variables: performance expectancy, efforts expectancy, social influence, and facilitating conditions. The first three are direct predictors of usage intent and behavior, whereas the fourth is a predictor of user behavior. The influence of the four major constructs on usage intention and behavior is thought to be moderated by gender, age, experience, and voluntariness of use. Authors created the theory by reviewing and combining the constructs of eight previous models used to explain information system usage behavior (theory of reasoned action, technology acceptance model, motivational model, theory of planned behavior, a combined theory of planned behavior/technology acceptance model, model of personal computer use, diffusion of innovations theory, and social cognitive theory) [9,10].

Researchers observed that UTAUT accounted for the variances in behavioral intention to use and actual use in a longitudinal study [9]. According to them, performance expectancy is a belief that narrates that technology will enable the attainment of desired improvements in task performance. Performance expectancy in e-shopping indicates the extent of a customer’s perceptions or views that e-shopping will facilitate them in attaining a precise performance level. The concept of performance expectancy is based on five sub-constructs: extrinsic motivation, perceived usefulness, outcome expectations, relative advantage and job fit. The main benefit, linked with performance expectancy, is a user’s productivity by saving time because they perceive that technology will enhance their performance.

Similarly, effort expectancy is defined as the level of effortlessness in utilizing systems or technology [9]. Since many customers regard e-shopping as effortless, perceived usefulness, complexity, and perceived ease of use can be studied under effort expectancy. As highlighted in the earlier research, these concepts are closely identical and are measured by the same scales [28]. The researcher also explored that effort expectancy influences user’s intention toward mobile banking technology [29]. As examined by authors, effort expectancy positively influences behavioral intention [30].

Likewise, social influence is described as the degree of perception of a user, based on people who are important to him, regarding the use of new systems/technology [9]. The customers’ perceptions are driven by important people (friends/colleagues) in an e-shopping context. Social influence is profoundly intricate and entails a variety of contingent influences. A person’s behavior is affected by social influence via three mechanisms: identification, compliance, and internalization [31]. These mechanisms are identified for compliance in the obligatory conditions that cause social influence to affect intention directly.

Finally, facilitating conditions are the extent to which a user believes that a firm’s infrastructure exists to support technological usage [9]. In the e-shopping context, facilitating conditions are the extent to which customers/shoppers perceive that they have the means to do e-shopping, including laptops, websites etc. This construct encapsulates three concepts: facilities, perceived behavioral control and compatibilities. Each concept is operational to measure firm or technology-related characteristics to eradicate the obstacles that may thwart its usage. Previous researchers have revealed a strong relationship between facilitating conditions and behavioral intention [32,33].

### 2.1. The extension of UTAUT

It was found during the literature review that UTAUT captures only a few aspects of the theory. In some cases, the role of the moderator is overlooked [34]. The researchers have expanded, incorporated, and used UTAUT across a range of organizational and technological contexts. For instance, researchers utilized it to explore mobile users’ (customers) technology use and adoption behavior [35]. Moreover, the authors also applied UTAUT to operational, middle, and senior-level employees to examine the technology use behavior [36]. Furthermore, the authors also analyzed citizens’ behavior to utilize e-government services [10].

Despite various wide-ranging applications, the extensions and replications of UTAUT are essential for identifying the acceptance of technology and extending its theoretical frontiers. For example, researchers have developed an extended form of the theory (UTAUT2) based on IT acceptance/usage behavior-related literature [10]. UTAUT2 recognizes more relevant antecedents/predictors and is considered a robust approach for understanding the central phenomenon of e-shopping behavior. In UTAUT2, new constructs were identified, such as hedonic price, habit, and motivation, while one moderator was dropped, i.e., voluntariness. Moreover, a link was added between facilitating conditions and behavioral intention. Furthermore, moderating relationships of age, experience, and gender were included.

The authors explained that UTAUT might have various extensions, such as adding new exogenous, endogenous, moderation, and outcome mechanisms [11]. Accordingly, in this study, an attempt has been made to extend UTAUT with one exogenous mechanism (e-shopping service quality) and one moderation mechanism (offline brand trust) between endogenous variables and e-shopping intention.

## 3. Hypotheses development

### 3.1. E-shopping Service Quality (OSSQ) and E-shopping Intention (OSI)

The existing research on service quality provides a deeper understanding of the factors influencing consumers’ service quality evaluation for various products/services. Accordingly, service quality is the customer’s assumption concerning the systems and services’ reliability, security, empathy, and credibility [37]. Authors claim that web-design elements (such as images, graphics, information, and video demos) are the prime characteristics of consumers’ decisions [38]. These elements need considerable improvements to increase market share in a highly competitive environment. The authors [39] stress the importance of interactivity and personalization on e-shopping websites, as they significantly impact service quality and customer experience for e-shopping [39]. Authors observed that navigation and shopping would be easy and enjoyable if a website is supportive and user-friendly [40]. In this way, customer’s reliance on a website can be built by enhancing the quality of services [41]. Accordingly, there is a greater likelihood of customer motivation if the quality of services is appropriately taken care of. Authors argue a powerful relation between quality of service and e-shopping acceptance [42]. Authors exploring the service quality of websites found that a consumer’s loyalty to the service provider is mainly dependent upon the high quality of service [43]. In the same vein, security, compared to the reliability, is the chief factor in hampering or contributing to inexperienced users’ intention for e-shopping [44].

Authors identify an (appealing) website being an essential aspect of e-shopping, which is generally used by online businesses for communication and interaction with online shoppers [45]. Researchers suggest that a customer’s quality judgment regarding e-retailers largely relies on the website service [46]. A study has also proposed betterments in website service quality to induce customer requirements over the retailer’s online website [47]. Likewise, understanding the website quality and the need for improvement from a consumer’s standpoint is required to deliver first-rate services. Similarly, a study substantiated the claim by citing evidence from the private banking sector where the quality services influence the customer’s behavioral intentions [48]. Therefore, the following hypothesis is posited:

H_1_: A significant relationship exists between E-shopping Service Quality and E-shopping Intention.

#### 3.1.1. E-shopping Service Quality and E-shopping Drivers

In virtual situations, where identical products are put on sale, competition becomes highly ferocious if it is only based on cost leadership strategy [49,50]. In this condition, several researchers have highlighted that online seller’s success, in the long run, depends on high service quality [51]. Resultantly, customer’s e-shopping drivers are likely to be impacted by service quality. Earlier research analyzed the content quality and website design [52,53]. For example, a better-designed website showing multiple shapes and dimensions of the product can increase customer’s browsing performance (e.g., 3D view). A trustworthy retailer will meet delivery expectations and adequately address security concerns regarding transactions. An online store will boost customer’s confidence by offering a high quality of service. Also, it has a range of communication channels for interaction and communication and addresses complaints or queries such as email, chat room, phone, and fax. A well-designed website is user-friendly, time-saving, and requires little mental effort (or energy saving) from online shoppers [54]. Researchers also ratify that user-friendly web design significantly impacts customers’ use of e-shopping [42].

On the other hand, authors also observed that user-unfriendly and complex websites or interfaces hinder shoppers’ intention to buy online [55]. E-retailers resolve such issues by making the shopping experience error-free and secure. According to the authors, a website graphical interface entails visualizing the product on the website. The customer’s purchase intention can be high if the template is good, the font is readable, and the user-friendly color scheme [20]. Picture quality and various product angles are also important elements of product visualization. Moreover, the anticipated ease of use affects customer’s intention to shop if the website responds /loads promptly, navigates fast, and offers better-searching options [21]. A good website is multilingual and provides different languages to choose from following customer’s areas and proficiency. Similarly, the interaction between customers and e-retailers is made easier through service quality.

The author argued that e-shopping drivers clarify why people use technological applications to achieve their goals [9]. So, e-shopping service quality can be a source to influence e-shopping drivers. Efficient e-shopping service quality is a significant feature for offering quality and flexibility to propel online shopping. Therefore, customer’s expectations and service quality are intertwined in e-shopping. Hence, the following hypotheses are proposed:

H_2_: A significant relationship exists between E-shopping Service Quality and Performance Expectancy.
H_3_: A significant relationship exists between E-shopping Service Quality and Efforts Expectancy.
H_4_: A significant relationship exists between E-shopping Service Quality and Social Influence.
H_5_: A significant relationship exists between E-shopping Service Quality and Facilitating Conditions.

### 3.2. E-shopping drivers and E-shopping intention

The researcher maintains that performance expectancy significantly influences behavioral intention [56]. There are many benefits of e-shopping for customers: shoppers can save time, make variety and price comparisons, and ask for product/services information. It was also being propounded that users’ cognitive benefits are represented by performance expectancy in using new technology [57]. These benefits have been chiefly regarded for users’ volition and sense of adopting heterogeneous technological applications [58]. Research suggests that behavioral intentions are affected by the cognitive and behavioral attempts to assimilate and employ an IT item (i.e., e-shopping), especially at the early stages of technological usage [59]. Users regard perceived effectiveness to embrace new technology; nonetheless, the more crucial aspect is the simplicity and user-friendliness of the system or technology (such as effort expectancy) [60,61]. Moreover, it was also observed that customers’ intention for e-shopping increases when they rely less on the company’s staff [62].

In the same vein, customers’ purchase intention will be high if they know that their relatives and friends also shop online or if they approve of e-shopping, and thus create social pressure [63]. Primarily, the customers are driven by the societal trends usually set by the family member(s) and friend(s) [64]. Consequently, these societal trends affect customer’s purchase intentions [63]. Hence, social influence has prompted customers to exchange their experiences and knowledge with others (e.g., social networking). Their estimation of these services and products may hold greater value than the luring of the manufacturers or providers. Such feedback is vital for consumers in making decisions to purchase [65,66].

Likewise, facilitating conditions (such as technology resources) are essential for the users to overcome barriers in using technology, particularly when technology is being adopted [67]. Afterward, facilitating conditions may affect the customer’s sense of convenience in technology. Therefore, an affirmative approach to the usage of devices and infrastructural support should alleviate the customer’s apprehensions about technology usage. Given the facilitating conditions, a customer is convinced that essential resources, knowledge, and help are at his disposal during online shopping. In UTAUT, for the use of IT artifacts, facilitating conditions are considered an aggregate of support. These rationales mentioned about e-shopping suggest that e-shopping drivers may cause intention to shop online. Hence, the following hypotheses can be formulated:

H_6_: A significant relationship exists between Performance Expectancy and E-shopping Intention.
H_7_: A significant relationship exists between Efforts Expectancy and E-shopping Intention.
H_8_: A significant relationship exists between Social Influence and E-shopping Intention.
H_9_: A significant relationship exists between Facilitating Conditions and E-shopping Intention.

### 3.3. The moderating role of offline brand trust

Brand trust can be interpreted as the customers’ readiness to bank on a particular brand, expecting it to function desirably and give favorable outcomes [68]. Offline brand trust designates a kind of trust or faith in the customer’s mind about the brand, born out of its offline purchase. A retailer can build its trust through its website, which can help reduce or weaken incredulities in a customer’s mind regarding e-shopping [69]. Research argues that purchase decisions are predicted by trust, both in offline and online modes. However, it is imperative in the latter’s case since interactions between a customer and a website without any human involvement. Thus, it is implausible that purchase transactions will happen without trust [70].

Authors also suggest that performance expectancy is significantly linked with purchases from the internet [56]. However, where the offline brand trust is low, such linkage becomes weaker. Brand trust in the e-shopping context is used to reduce consumers’ negative perceptions about e-shopping [69]. Subsequently, offline brand trust is vital in reducing customers’ adverse intentions concerning the offline product(s) or service(s). Since online buying systems entail various uncertainties at different levels, particularly when customers’ credentials or any other information (e.g., credit card) are required, customers’ trust in retailers can reduce suspicions about e-shopping. When this happens, customer performance goes high. The authors reported an important connection between effort expectancy and behavioral intention [10]. Offline brand trust can boost and strengthen this connection since it depends on brand reputation and predictability. A reputable brand is easy to buy because customers think it must facilitate e-shopping. Similarly, brand predictability encourages confidence because the customers will be sure about the safety of their transactions, and the purchase will become easy.

Thus customers’ online purchasing value is affected by their offline perceptions and experiences, ultimately determining their online purchase intentions. One way to create such value is to propagate/publicize up-to-date technology information among important people, i.e., colleagues, friends, relatives. Different social platforms or communication means can be used to disseminate this information. Marketing literature acknowledges “Customer Trust” as a critical factor for business success and building longstanding customer relationships. In offline brand trust, the belief holds a value that people shall have similar confidence levels for traditional and online stores. In a particular situation where customers are suspicious of e-shopping, their trust will probably be inclined toward a retailer’s website [68].

Therefore, in scholarly research, longstanding relationships and constant communications are considered crucial factors in trust-building [71]. The value-creating processes and practices, including customer-product, and brand-market, are the brand community parts [72]. Owing to social media, the brand community can help establish durable ties and intimate contacts without losing or harming connections built offline [73]. Likewise, a product can be perceived as risky for several reasons, such as uncertain facilitating conditions [9]. Customers can also be confounded, doubting whether a product can facilitate to gain their desired response or not. But if a customer feels that other customers (or people) think that the brand is good, he will trust the brand [19]. Also, he will be delighted to share such experiences with friends and family, even with low e-shopping drivers. Furthermore, he cannot blame the brand and will not be confronted by any direct peer-objection or peer-criticism. By this, high offline brand trust develops a relation between e-shopping drivers and e-shopping intentions. Consequently, the following hypotheses have been proposed:

H_10_: The relationship between Performance Expectancy and E-shopping Intention will be stronger when Offline Brand Trust increases.
H_11_: The relationship between Efforts Expectancy and E-shopping Intention will be stronger when Offline Brand Trust increases.
H_12_: The relationship between Social Influence and E-shopping Intention will be stronger when Offline Brand Trust increases.
H_13_: The relationship between Facilitating Conditions and E-shopping Intention will be stronger when Offline Brand Trust increases.

### 3.4. E-shopping intention and E-shopping adoption

Scholars have shown great interest in the behavioral intention for online shopping. Various e-shopping related theoretical models have been employed to measure the intention and the actual behavior of customers. Researchers reveal that behavioral intention correlates with actual behavior [10]. Therefore, an assessment of the customer’s intentions would offer appropriate signs about the behavior. Researchers described that behavioral intention changes the intention and actual behavior [9]. Besides, various scholars e.g., [74,75] have provided evidence of a direct connection between intention and actual behavior. Moreover, the intention is established as a critical component for actual customer use and functions as a surrogate to assess the actual behavior [76]. Researchers also examined the customers’ behavioral intention and found that users’ intention can determine mobile app technology adoption [77]. Earlier researchers also examined intention as a vital element determining actual use in 3G technology and intention affects a user’s behavior [47,78]. Hence, the following hypothesis is proposed:

H_14_: A significant relationship exists between E-shopping Intention and E-shopping Adoption.

**Fig 1:**
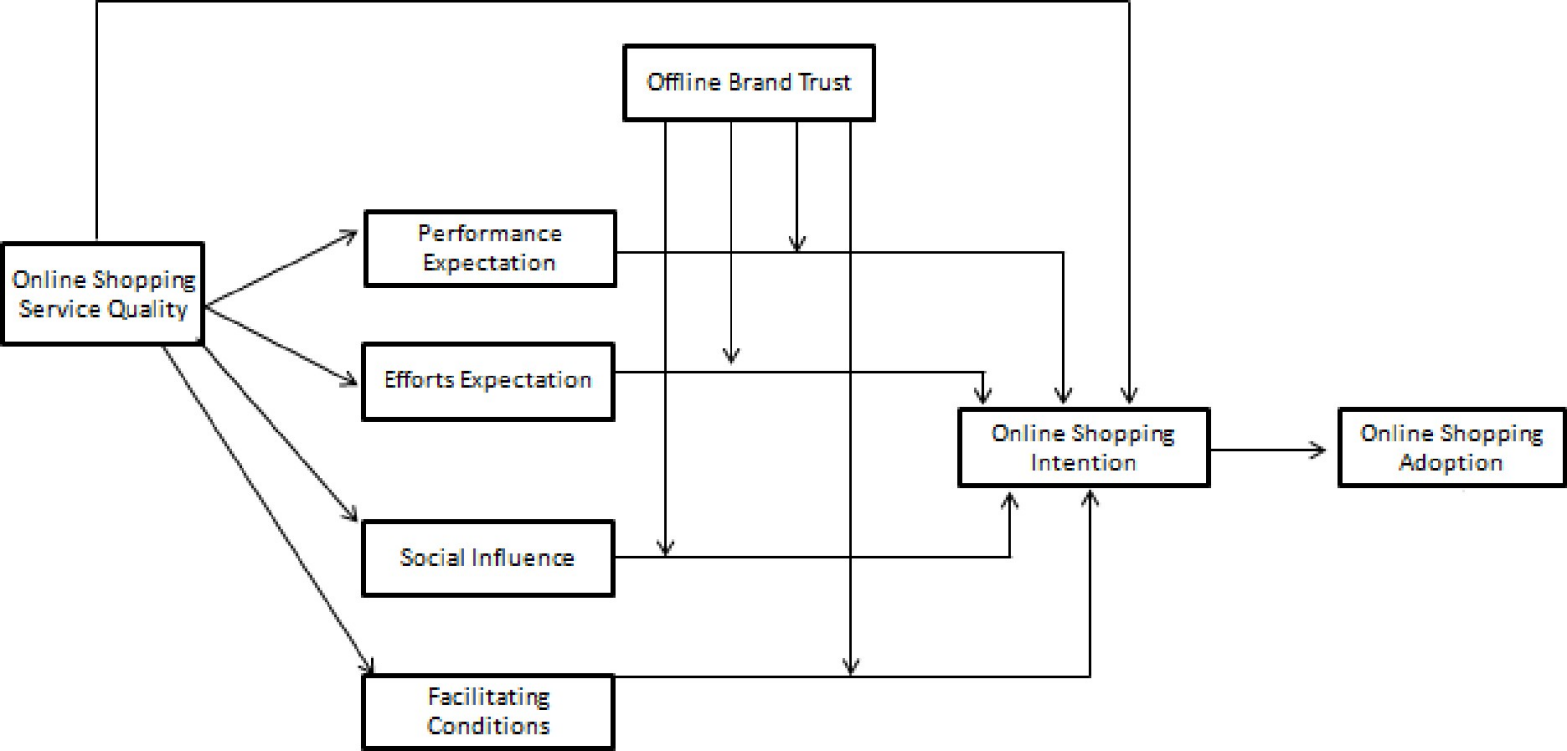
The hypothesized relationships.

The preceding hypothesized relationships have been depicted in Fig 1.

## 4. Methodology

### 4.1. Sample and procedure

Since much of the existing literature in a similar context has come from the developed countries in the west, research from Pakistan can endow with a more worldwide view. Pakistan is an emerging country located in South Asia with a strong presence in the e-shopping market. Therefore, e-shoppers of Pakistan (i.e., target population) were contacted to conduct a full-scale administrative survey (using quantitative nature and positivism philosophy of research).

A trial run on 38 e-shoppers in Pakistan was conducted prior to the survey’s launch. Cronbach’s alpha test was used to assess the test-run, and it came back at 0.913, indicating that the survey was ready for full-scale administration. Later on, the services of a well-reputed panel company (a company that matches the respondents with the target audiences for a fee purpose) in Islamabad (the capital city of Pakistan) were utilized to conduct the survey. The survey was accompanied by a cover letter explaining the research objectives and assurances of the confidentiality of the participant’s responses. This study’s cross-sectional nature reduced the likely biases in data collection by gathering it in four time-lags (an in-compliance approach with the study [79]). Each questionnaire was given a code, and a proper record was maintained to link the responses of the same respondent at different points in time.

The planned time lag was around four months for four phases. Each phase was implemented after 21 days from the implementation of the previous phase. In the first phase, the data for ‘online shopping service quality’ were collected in 14 days. The data for ‘online shopping drivers’ and ‘offline brand trust’ were gathered in the next 14 days in the second phase. While, in the third phase, the data for ‘online shopping intention’ was collected in the following 14 days. Finally, in the fourth and last phase, the responses for online shopping adoption were obtained in the succeeding 14 days.

A total of 1000 Pakistani e-shoppers were invited to participate in the survey. Out of them, 403 responded. On initial screening, the survey response from 47 participants was declared invalid due to missing data and extreme outliers. Consequently, the valid sample size was turned out as 356, with an effective response rate of 35.6%. According to the authors, a sample size of more than 200 is considered adequate, while a response rate above 25% is regarded as good [23,25,26,80].

The demographics (Table 1) show that 68.8% were male, whereas the age category indicates that 31.7% of the respondents were less than 20 years old, 53.7% had an age between 20 to 30 years, and 14.6% were from 31 to 40 years. The table also presents the educational level of the respondents: 39.3% were below graduates, 59.3% were graduates, and only 1.4% of respondents were above graduates. In addition, the monthly household income of 21.3% of the participants was below US$ 150, 57.5% were between US$ 150 and 400, and 21.2% was above US$ 400. Furthermore, concerning the shopper’s online experience, 9% had below six months, 22.8% had between six months to a year, 41.3% had above year but below two years, and 27% had above two years. Lastly, 63.3% of respondents often buy or look for fashion as e-shopping merchandise, and 10.2%, 11.1%, 6.2%, 3.1%, and 3.1% buy or look for electronics and media, food and personal care, toys and hobby, furniture and appliances, and others respectively.

**Table 1:** Demographics of the respondents.

Characteristics	Categories	Percentage
Gender	Male	68.8
	Female	31.2
Age	Less than 20 Years	31.7
	20–30 Years	53.7
	Above 30 Years	14.6
Education Level	Below Graduate	39.3
	Graduate	59.3
	Above Graduate	1.4
Household Income (Monthly)	Below US$ 150	21.3
	US$ 150–400	57.5
	Above US$ 400	21.2
E-shopping Experience	Below 6 months	9.0
	6 months to 1 year	22.8
	1 year to 2 years	41.2
	Above 2 years	27.0
E-shopping Merchandise	Fashion	66.3%
	Electronics and Media	10.2%
	Food and Personal Care	11.1%
	Toys and Hobby	6.2%
	Furniture and Appliances	3.1%
	Others	3.1%

Overall, the practice of hiring the services of panel company turned out to be extremely useful not just because of receiving a reasonable response rate, but because the gender and e-shopping merchandise parameters of the respondents were observed to be in line with the reports of PRNewswire (cited by [25]). All the demographics of the respondents are summarized in Table 1.

### 4.2. Ethics statement

Ethical review and approval were not required for this study on human participants following the local legislation and institutional requirements. Moreover, the participants provided their written informed consent to participate in this study.

### 4.3. Measures

For all the variables except E-shopping Adoption (OSA), a five-point Likert scale (1 = strongly disagree to 5 = strongly agree) was utilized to obtain the responses. OSA was measured on a scale anchoring 1 = once in two years to 5 = once in a week. The measures were adopted from various studies described below, and the items have been presented in Table 3.

E-shopping Service Quality (OSSQ) was computed through 5 aspects: website design, reliability, responsiveness, communication, and trustworthiness. Three factors (website design, reliability, and responsiveness) were adopted [18,42]. The other two aspects (trustworthiness and communication) were adopted from existing literature [18,81]. The reported aggregate of α value is 0.814.

The researchers developed Performance Expectancy (PE), Efforts Expectancy (EE), Social Influence (SI), and Facilitating Conditions (FC) [9]. Several recent scholars have used this construct [10,82]. The similar has been adapted within the context of online shopping. The analysis reported α reliability of 0.88, 0.78, 0.92, and 0.85, respectively.

Offline Brand Trust (OBT) was adopted from [61], and measured through two aspects: brand predictability and brand reputation. The reported aggregate of α value is 0.89. E-shopping Intention (OSI), developed [61], was also used by [9]. The same has been adopted within the e-shopping context. The alpha reliability reported in the original survey is 0.82. Lastly, E-shopping Adoption (OSA) was measured based on the frequency of e-shopping by the respondents [83].

## 5. Data analysis and results

Authors suggest that common method bias (CMB) can be attributed to the spurious variance in measuring the constructs due to the study’s cross-sectional nature [84]. CMB is one of the prime sources that negatively influence the validity of empirical findings [85]. Harman’s Single Factor test is widely used to measure CMB in the self-reported data [86]. We employed this test by conducting un-rotated exploratory factor analysis (EFA) using SPSS to determine the number of factors that account for the variance in the variables. The results showed that the single factor explained only 14% variance. As a standard, if the variance is <50%, the research data have no CMB issue [84].

Then we computed mean, standard deviation, skewness and kurtosis for all the latent constructs as displayed in Table 2. Mean values show the essence of the customers’ observations about a particular variable. The mean values range from 2.5033 to 3.9110. The values greater than the midpoint (2.5) indicate positive behavior. The range of standard deviation is from 0.70661 to 1.07065, implying that the data are less scattered. Furthermore, skewness and kurtosis values demonstrate that the data were normally distributed (threshold of -1 to +1.0) [87].

**Table 2:** The descriptive statistics.

Variables	N	Mean	Std. Deviation	Skewness		Kurtosis	
				Statistic	Std. Error	Statistic	Std. Error
OSSQ	356	2.5033	.82836	.707	.129	.204	.258
PE	356	3.7346	.97546	-.873	.129	.128	.258
EE	356	3.1088	.94193	-.088	.129	-.518	.258
SI	356	3.1021	1.04539	-.200	.129	-.919	.258
FC	356	3.0330	1.04686	-.214	.129	-.908	.258
OSI	356	3.0225	1.07065	-.421	.129	-1.000	.258
OBT	356	3.9110	.70661	-0.502	.129	1.984	.258

Afterwards, we performed the confirmatory factor analysis (CFA) to confirm the constructs’ distinctiveness using AMOS-24. The revised model after deleting one item from each of OSSQ and OBT provided good fit to the data (*χ*^2^/df = 1.879; IFI = 0.936; TLI = 0.931; CFI = 0.936; RMSEA = 0.050; SRMR = 0.0390) [88,89]. In addition, the reliability was measured through Cronbach’s alpha value (0 to 1). In a study, the author’s point of view is that a minimum alpha value of 0.7 demonstrates good reliability [90]. Table 3 illustrates that the “α” values of all the variables were above the threshold. In addition to reliability, validity is also required to compute the goodness of a construct. The construct validity was assessed through face validity, convergent validity and discriminant validity. Face validity was demonstrated because all measures were adopted from previous studies. Convergent validity measures the extent to which items are correlated. It was established because the loading values of all items were above 0.5 and significant at p<0.001, as shown in Table 3.

**Table 3:** The items and their factor loadings.

Constructs & Measurement Items	Standardized Factor Loadings
**Online Shopping Service Quality (OSSQ)**	
E-stores are visually appealing and attractive.	.788
Completing an e-shopping transaction is easy and quick.	.816
When e-retailers promise to do something, they exactly perform as promised.	.753
While solving the issues of customers, sincerity is shown by the e-retailers.	.866
The transactions from e-stores are error-free.	.873
E-stores have adequate security for online transactions.	.769
The online stores give prompt services to the customers.	.761
E-retailers are always willing to help the customers.	.719
E-stores are never too busy responding to their customers’ requests.	.761
I feel secure while providing sensitive information for e-purchases.	.856
I feel a low risk of e-purchasing.	.956
I can depend on the e-retailers.	.776
The purchase from the e-retailer is good buying.	.825
E-stores often provide their contact details.	.740
E-stores often provide their customer chat services.	.708
Customer service representatives of e-stores can conveniently be contacted.	.760
E-stores often offer a variety of language (e.g., Urdu/English) options	.708
The “Frequently Asked Questions” page often addresses my questions/queries/problems.	.770
**Offline Brand Trust (OBT)**	
Reputed Brands are good.	.756
Reputed Brands are reliable.	.775
Other people believe that brands are not good.	.773
Other people believe that brands are reliable.	.867
Brands are reputed in terms of their performance.	.828
Concerning brands, I have heard negative comments.	.760
I know my expectations while purchasing the brands.	.763
I often anticipate accurately how the brands will perform.	.740
Brands perform consistently.	.709
I’m not sure how the brand will perform next time.	.775
Brands can always be counted on to my expected performance.	.760
**Performance expectancy (PE)**	
E-shopping is useful in my daily life.	.853
E-shopping helps me to find the required products in a very shorter time.	.843
I can find some products/services online that are not available in physical stores.	.846
Using e-shopping can increase my efficiency.	.828
**Effort Expectancy (EE)**	
E-shopping procedure is easy to learn.	.806
My interactions with e-shopping are understandable.	.761
I find e-shopping easy to use.	.879
It is easy to become skillful in e-shopping.	.806
**Facilitating Conditions (FC)**	
I have the necessary resources to use e-shopping.	.896
I have enough knowledge of e-shopping	.725
E-shopping is well-matched with the technologies I use (e.g., Facebook).	.822
People are conveniently accessible to help me use e-shopping websites whenever I feel difficulties.	.797
**Social Influence (SI)**	
The important people in my life think that I should shop online.	.804
People who inspire my behavior think that I should shop online.	.883
People whose opinions are important to me think that I should use online shopping.	.837
**E-shopping Intention (OSI)**	
In the coming month(s), I intend to use e-shopping.	.886
In the coming month(s), I predict that I will use e-shopping.	.875
In the coming month(s), I plan to use e-shopping.	.867

CFA (*χ*^2^/df = 1.879; IFI = 0.936; TLI = 0.931; CFI = 0.936; RMSEA = 0.050, SRMR = 0.0390).

The average variance extracted (AVE) values were above the threshold value of 0.5 [91], as exhibited in Table 4. Moreover, discriminant validity measures the extent to which items contribute uniqueness to their respective construct. Since the most commonly used Fornell-Larcker criterion to determine discriminant validity has been met with criticism. For example, the authors also argued that the said criterion is not able to detect the absence of discriminant validity in routine research contexts. Thus, another technique was employed based on the multitrait-multimethod matrix, known as Hetero Trait and Mono Trait (HTMT). Since the computed values of HTMT are less than the threshold of 0.85 [92], it demonstrates discriminant validity, as shown in Table 4.

**Table 4:** HTMT analysis for discriminant validity.

	AVE	OSSQ	OBT	PE	EE	FC	OSI	SI
OSSQ	0.627							
OBT	0.599	0.057						
PE	0.710	0.352	0.024					
EE	0.663	0.371	0.054	0.606				
FC	0.659	0.362	0.055	0.540	0.571			
OSI	0.767	0.502	0.139	0.638	0.678	0.669		
SI	0.709	0.475	0.063	0.570	0.588	0.542	0.726	

After confirmation of construct validity, the next step is structural equation modeling (or path analysis). It tends to the likely explanation of variance in the endogenous variable due to the direct/indirect influence of exogenous variables. The path analysis proved goodness of fit (*χ*^2^/df = 2.687; IFI = 0.911; TLI = 0.903; CFI = 0.910; RMSEA = 0.069) [89]. The estimates and significance of the direct effects of the constructs have been shown in Table 5. The result of H_1_ justifies this hypothesis (β = 0.596, p < .05). Moreover, the results of H_2_, H_3_, H_4_, and H_5_ support these hypotheses (β = 0.538, p<0.001; β = 0.460, p<0.001; β = 0.596, p<0.001 respectively). Furthermore, the results of H_6_, H_7_, H_8_ and H_9_ provides the support for these hypotheses (β = 0.163, p<0.001; β = 0.265, p<0.001; β = 0.346, p<0.001; β = 0.235, p<0.001, respectively). Finally, it was checked whether e-shopping intention further prompts to adopt e-shopping (H_14_). The results (β = 0.239, p<0.001) validated the hypothesis.

**Table 5:** Direct effects of the structural model.

Hypothesis	Relation	Estimate	S.E.	C.R.	*p-v*alue	Result
H_I_	OSI ← OSSQ	0.596	0.072	8.289	0.039	Accept
H_2_	PE ← OSSQ	0.538	0.082	6.539	***	Accept
H_3_	EE ← OSSQ	0.460	0.070	6.575	***	Accept
H_4_	SI ← OSSQ	0.596	0.072	8.289	***	Accept
H_5_	FC ← OSSQ	0.513	0.080	6.418	***	Accept
H_6_	OSI ← PE	0.163	0.037	4.416	***	Accept
H_7_	OSI ← EE	0.265	0.046	5.784	***	Accept
H_8_	OSI ← SI	0.346	0.049	7.060	***	Accept
H_9_	OSI ← FC	0.235	0.038	6.133	***	Accept
H_14_	OSA ← OSI	0.222	0.048	4.632	***	Accept

***p < 0.001.

SPSS 26 was used to perform descriptive and moderation analyses through Process macro 3.5 [93]. Moderation analysis was conducted to investigate Offline Brand Trust (OBT) as a moderator between OSDs and OSI such that the effect will be stronger with high OBT than its low value. We utilized model 1 of Process macro for the moderation analysis. The results for all moderating effects have been described in Table 6.

**Table 6:** Results of moderating effects.

Path		Coeff.	t	LLCL	ULCL	Statistic	Results
*PE*→*OSI*	Performance Expectancy	0.2072	3.6004	0.0940	0.3204	*R*^2^ = 0.1202 *F* = 16.0230 *p* = 0.0000	Supported
	Offline Brand Trust	0.1574	1.9573	-0.0008	0.3155		
	Interaction	0.3386	4.2551	0.1821	0.4952		
*EE*→*OSI*	Efforts Expectation	0.3397	6.0834	0.2299	0.4495	*R*^2^ = 0.1600 *F* = 22.3496 *p* = 0.0000	Supported
	Offline Brand Trust	0.2161	2.9143	0.0703	0.3620		
	Interaction	0.3109	4.2528	0.1671	0.4547		
*SI*→*OSI*	Social Influence	0.5688	12.6620	0.4804	0.6571	*R*^2^ = 2324 *F* = 57.4223 *p* = 0.3791	Not Supported
	Offline Brand Trust	0.1654	2.3807	0.0288	0.3020		
	Interaction	0.0524	.8806	-0.0647	0.1695		
*FC*→*OSI*	Facilitating Conditions	0.4988	10.7016	0.4071	0.5905	*R*^2^ = 0.2695 *F* = 43.2768 *p* = 0.0079	Supported
	Offline Brand Trust	0.2110	2.9977	0.0726	0.3495		
	Interaction	0.1538	2.6735	0.0407	0.2670		

The main effects of all the predictors are significant (t>1.96; [LLCL—ULCL]≠0). This follows the expectation that e-shoppers perceived online shopping drivers (PE, EE, SI and FC) leading to online shopping intention. The interaction terms of the three effects, i.e., PE, EE, and FC are significant, and the intervals do not contain zero. Therefore, we conclude that offline brand trust moderates the majority of the relationships. The magnitudes of interacting effects and intervals of PE, EE and FC with OBT are (β = 0.3386; LLCL = 0.1821, ULCL = 0.4952), (β = 0.3109; LLCL = 0.1671, ULCL = 0.4547) and (β = 0.1538; LLCL = 0.0407, ULCL = 0.2670), respectively. However, OBT could not moderate the relationship between SI and OSI (β = 0.0524; LLCL = -0.0647, ULCL = 0.1695). In other words, Performance Expectancy, Efforts Expectancy and Facilitating Conditions make Online Shopping Intention when consumers have greater Offline Brand Trust.

**Fig 2:**
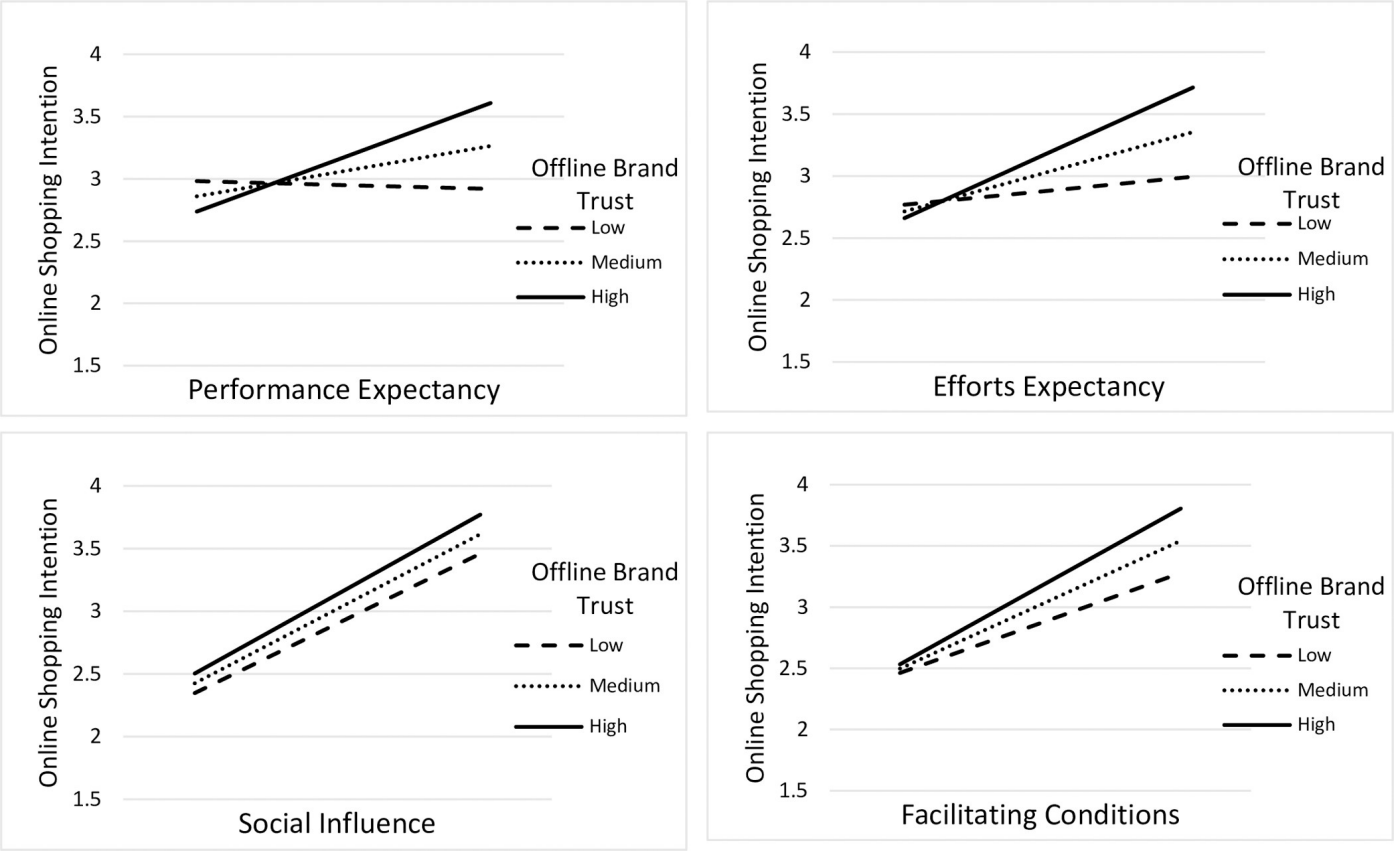
Moderating effect of offline brand trust between online shopping drivers and OSI.

The graphs have also been plotted in Fig 2 to visualize the moderating effects. The relational lines are inclined toward the horizontal axis when online brand trust is low but oblique when it is effectively ingrained among online shoppers. It can be seen that online brand trust induces the strongest online shopping intention due to performance expectancy, whereas it could not cause intention because of social influence in the local context.

## 6. Discussion

This study analyzes online shopping thoroughly, identifying the leading elements that foresee the customers’ decision to utilize such systems. The study was designed to understand the significant factors that can contribute to the choices made by online consumers, such as e-shopping service quality, e-shopping intention, e-shopping adoption, offline brand trust, performance expectancy, efforts expectancy, social influence, and facilitating conditions. The outcomes of this research are consistent with the existing literature. The researcher noted that an online retailer with a website high in quality in terms of its appearance and performance generates positive attitudes of customers [94]. Furthermore, the authors also found that a service provider can gain a customer’s loyalty by providing exceptional service [43]. The author suggests that the quality of services can substantially positively affect behavior [48]. This new research supports the previous studies which show a correlation between service quality and the drivers of e-shopping [54,95]. The study hypothesized that the service quality of e-shopping would predict the drivers of online shopping, and the results support this hypothesis. Therefore, it is beneficial for the retailers to provide a high-quality e-shopping experience, such as an easy-to-use website and a friendly user interface, which does not require much effort from the customer for product selection.

This study also found an important relationship between e-shopping drivers and e-shopping intention. These findings are also supported by many researchers [9,57,56,96]. The study also found that the perception of a customer toward technology, being useful, helps them perform their daily tasks and activities [9]. It also saves time and effort and increases their productivity. Certain types of e-shopping drivers (such as effort expectancy and performance) can signify the perceived cognitive interests of new technology usage [57]. Such benefits can be very important in influencing a customer’s perception in adopting new and different types of technologies [58]. Therefore, significant features like saving time, comparison between many different products, and information about products and services are excellent sources to create the intention of e-shopping among the consumers.

This research shows the positive influence of e-shopping drivers and how it can significantly impact e-shopping intention. However, the role of brand trust must also be noted since it can greatly help decrease the perceived risk to impulse customer’s purchase intention. These findings show that offline brand trust can affect three e-shopping drivers: facilitating conditions, performance expectancy, and efforts expectancy. A consumer’s positive experience with a conventionally operated retailer can be very important in e-shopping from the same retailer. According to research, a consumer’s trust gained through conventional offline stores significantly impacts and predicts their confidence in online shopping [69]. Therefore, consumers will confidently and comfortably shop from an online retailer with high trust [97,98]. According to a research study, a consumer’s willingness to continue shopping online increases with a feeling of security [99].

The offline brand trust did not moderate the effect of social influence on the intention of shopping online. This contradicts the research, which states that social influence plays a significant role in constructing a consumer’s perception of the use of the technology [99]. Another study supports this concept and states that social norms are an essential aspect of an individual’s decision to adopt technology or reject it [100]. However, some consumers may have uncertainty regarding products and services due to a multitude of reasons. For instance, consumers might be uncertain about a product’s performance [101], or they may be uncertain about the social risk (whether owning or using a specific product would produce the desired response from their social network). In cases such as those mentioned above, the offline brand trust would not work as a source of moderation. Therefore, if the consumer believes that others think of the brand positively and believe it performs well and does not have any social risk, he may trust that brand, but it is implausible to advance the intention of online shopping.

The research ultimately discovered that the intention of e-shopping is what leads to e-shopping adoption. So, it is essential to learn which motivational elements can create an intention of online shopping, specifically in consumers that are prepared to engage in such behavior. Previous research, e.g., [74,75], has confirmed a direct relationship between intention and behavior. The researcher noted that intention is an excellent indicator of willingness to partake in a particular type of behavior and how much that behavior is performed [102]. Literature also explored behavioral intention and concluded that it is indeed an excellent predictor of actual behavior from a customer, which can be used as a proxy for measuring a customer’s behavior [103]. It has also been proved a proxy to measure users’ behavior [77].

## 7. Implications

As e-shopping has become more popular, organizations strive to achieve competitive advantages via e-businesses. The advancement in internet technology and computer optimization has improved the popularity of e-shopping worldwide [104]. This rapid development and growth of such e-commerce are introducing new models for conducting an online business. Considering the level at which consumers rely upon online shopping, there is a significant need to understand e-shopping behavior, especially in developing countries.

In this regard, this study contributes more toward the existing body of research information. By utilizing the e-shopping drivers, this study has examined the relationship between the quality of e-shopping service and e-shopping adoption. Moreover, through this study, an exogenous variable (i.e., e-shopping service quality) has been added for the first time in UTAUT. Furthermore, this study is pioneered by adding “offline trust” as a moderation mechanism into UTAUT. As a result, the challenge for online retailers to sell over the internet can better be tackled by enhancing e-shopping service quality and building offline label trust.

This study would help e-dealers, entrepreneurs, and online retailers because it has expanded the basics of UTUAT by putting in new variables. The new model has been refined and validated through survey data collected from Pakistani online shoppers. It will provide a global view because most of the current literature in the same context comes from Western countries.

Moreover, this study provides a thoughtful understanding to help online retailers enhance their consumer’s trust and e-shopping adoption. Since website service quality is essential in online shopping, building an easy-to-use website with striking web pages is needed. As highlighted, the importance of service quality encourages e-marketers to design a website, which eventually fulfills their customers’ requirements. Given that, online retailers are encouraged to improve the service quality of their websites. Research findings also suggest that online store drivers are critical. As a result, retailers should manage online consumers’ effort expectancy by making their website handy. Besides, they must assist in the form of technical support and infrastructure.

## 8. Conclusion

Due to the fact that expanding our understanding of customer behavior literature by adding additional exogenous and moderating variables to the UTAUT framework is a relatively unexplored field, this study endeavor proved to be both distinctive and valuable. As a result of the foregoing rationales, our objective was to investigate the relationships between e-shopping service quality (an exogenous variable), e-shopping drivers (performance expectancy, effort expectation, social influence, and facilitating conditions), e-shopping intention, and e-shopping adoption, with an emphasis on the moderating role of offline brand trust in an e-shopping context.

Structure equation modeling was used to verify the variable distinctiveness and path analysis. A sample size of 356 online shoppers in Pakistan was used. The results of this study indicate that e-shopping drivers are indeed influenced by e-shopping service quality. Likewise, it also demonstrates that e-shopping drivers lead to e-shopping intention and eventually to the adoption of online shopping. Furthermore, offline brand trust is the key factor that moderates the relation between e-shopping drivers and e-shopping intention. The data were collected in four phases to lower the possible effects of common methods biases.

This study presents a thorough understanding of online retailers so that consumers may adopt online shopping. Online retailers should aim to improve the service quality of their websites so that the purchasing intention of their customers can increase. The relationship between e-shopping drivers and e-shopping intention suggests that retailers need to fulfill the effort expectations of online shoppers. The website should have a friendly user interface, be easy to navigate, and should be able to meet the efforts expectancy of their customers. If the owners or the management of the retailers want their customers to shop online, they must provide exceptional technical support to their customers while they perform online transactions.

Although the research has numerous strengths, it has some limitations and directions for future studies. For instance, qualitative methods and longitudinal techniques should be used in future studies to understand the online shopping phenomenon further. Moreover, experimental studies should be conducted to explore customer behavior additionally. Furthermore, other constructs should be applied in future studies, such as user interface, technophobia, customer engagement, and community engagement.

In addition, this study has concentrated on the overall aspects of e-shopping without specifying the product category. Besides, e-consumers were selected as the standard sample of this study. It is, therefore, recommended to conduct future research that may evaluate the customer purchase behavior about a particular product (clothing, toys, support materials, electronics, etc.) because the customer’s purchasing targets vary in different product categories. Future studies can also collect data based on particular online shoppers’ demography, such as age, gender, or income levels.
